# Pharmacological approaches to understanding protein kinase signaling networks

**DOI:** 10.3389/fphar.2023.1310135

**Published:** 2023-12-14

**Authors:** Elloise H. Stephenson, Jonathan M. G. Higgins

**Affiliations:** Faculty of Medical Sciences, Biosciences Institute, Newcastle University, Newcastle uponTyne, United Kingdom

**Keywords:** kinome, kinase, kinase inhibitor, cell signaling, chemo-genetics, phosphoproteomics, systems biology, drug development

## Abstract

Protein kinases play vital roles in controlling cell behavior, and an array of kinase inhibitors are used successfully for treatment of disease. Typical drug development pipelines involve biological studies to validate a protein kinase target, followed by the identification of small molecules that effectively inhibit this target in cells, animal models, and patients. However, it is clear that protein kinases operate within complex signaling networks. These networks increase the resilience of signaling pathways, which can render cells relatively insensitive to inhibition of a single kinase, and provide the potential for pathway rewiring, which can result in resistance to therapy. It is therefore vital to understand the properties of kinase signaling networks in health and disease so that we can design effective multi-targeted drugs or combinations of drugs. Here, we outline how pharmacological and chemo-genetic approaches can contribute to such knowledge, despite the known low selectivity of many kinase inhibitors. We discuss how detailed profiling of target engagement by kinase inhibitors can underpin these studies; how chemical probes can be used to uncover kinase-substrate relationships, and how these tools can be used to gain insight into the configuration and function of kinase signaling networks.

## 1 Introduction

The clinical success of numerous protein kinase inhibitors has highlighted the importance of kinase signaling in disease (Cohen et al., 2021). It is clear that kinases function within complex signaling networks (Hopkins, 2008; Knight et al., 2010), and a better understanding of these networks will enable disease research and drug development. Pharmaceutical companies remain focused on well understood targets, and comprehensive characterization of kinase action within their networks in cells is needed to allow drug development to be pursued with a lower risk of failure (Oprea et al., 2018).

To fully understand kinase signaling in disease we must learn about the properties of the wider network in affected cells. For example, mutations or changes in expression level of one kinase are likely to invoke changes in the nature of other signaling pathways within the network that may contribute to the disease phenotype (Creixell et al., 2012). Indeed, it has been argued that increasing knowledge of signaling networks represents the most efficient approach to the development of new treatment options in cancer (Yaffe, 2013). Of course, an appreciation of how kinase signaling networks operate in normal cells will also be necessary to understand what changes in disease.

Importantly, drug efficacy is crucially dependent on the status of the pre-existing kinase networks in target cells. For instance, the growth of different tumors may be dependent on different components of signaling pathways, even in patients with apparently similar cancers (Mellinghoff et al., 2005). If the full potential of personalized medicine is to be fulfilled, we will need to characterize the status of signaling networks in individual patients and identify suitable diagnostic and prognostic disease biomarkers, as well as effective treatments (Knight et al., 2010; Graves et al., 2013; Cohen et al., 2021; Rocca and Kholodenko, 2021).

Many kinase inhibitors have been designed largely with a “one target—one drug” approach. However, signaling networks tend to be resilient and are often relatively unaffected by the inhibition of only one element (Hopkins, 2008; Knight et al., 2010). Indeed, the effectiveness of some drugs (e.g., sorafenib, cabozantinib) appears to rely on “off-target” activity of the agent in addition to the designed “on-target” activity (Wilhelm et al., 2006; 2004; Markowitz and Fancher, 2018). This reinforces the view that, in many cases, we may need to inhibit the activity of more than one type of kinase to obtain desired therapeutic effects. Therefore, another aim of systemic studies is to aid the identification of multiple sites for intervention in kinase networks to produce meaningful clinical responses.

Network pharmacology also aims to understand how cells respond to drug treatment. For example, during drug development, it is important to determine the on and off-target activity of kinase inhibitors in cells, and to understand the network-wide effects of drug action so that efficacy and potential side effects can be assessed. In some notable cases (e.g., imatinib), the identification of “off-target” activity of kinase inhibitors has led to drug repurposing and approval for use in additional conditions (Demetri et al., 2002).

Studies of networks should also help us understand how, particularly during cancer therapy, cells become resistant to kinase inhibitor drugs. Kinase mutations can directly prevent drug binding to the intended target but, more relevant here, signaling networks can also be rewired to evade drug action. For example, the activity of alternative kinases such as MET can circumvent the inhibition of EGFR by gefitinib (Engelman et al., 2007), and bypass pathways also occur upon inhibition of BRAF or CDK4/6 (Johannessen et al., 2010; Nazarian et al., 2010; Marusiak et al., 2014; Goel et al., 2022). We therefore need to be able to unravel the cellular signaling pathways that underlie drug resistance so that alternative therapies can be developed and made available to patients (Knight et al., 2010; Cohen et al., 2021; Rocca and Kholodenko, 2021). This highlights again an important wider point: kinase networks are plastic and context-dependent. The kinases that phosphorylate particular substrates may vary from cell type to cell type, or even in response to different stimuli. For example, either PKA or RSK1 can phosphorylate LKB1 Ser-431 depending on the stimulus (Sapkota et al., 2001). This must be considered in the development of methods to map kinase networks.

A wide array of technologies has been employed with the aim of answering these questions. There are many examples of non-pharmacological approaches to understand kinase-substrate relationships (KSRs) and kinase network topologies, such as genetic manipulation of cells and *in silico* predictions. Nevertheless, as pharmaceuticals remain the primary means of intervention in disease, it makes sense to embed pharmacological methods in early stage discovery efforts (Moellering and Cravatt, 2012), and these methods also have some significant benefits. Here, we will focus on the major pharmacological technologies that have been employed to elucidate kinase signaling networks in health and disease, and their advantages and disadvantages compared to other approaches.

## 2 Selectivity *versus* efficacy: chemical probes *versus* kinase inhibitor drugs

Cell-permeable small molecule kinase inhibitors are key pharmacological tools for laboratory studies of cellular kinase networks, and for therapeutic intervention in kinase signaling pathways in patients. In other words, small molecule inhibitors can be used as chemical probes or as kinase inhibitor drugs (or both). It is important not to lose sight of the differences between these two types of application. A chemical probe is “a selective small-molecule modulator of a protein’s function that allows the user to ask mechanistic and phenotypic questions about its molecular target in biochemical, cell-based or animal studies” (Arrowsmith et al., 2015). Knowing the pattern of target engagement and specificity of action is vitally important for a chemical probe, since the underlying assumption is often that the function of only a single target protein is altered. Unknown off-target activity of chemical probes severely limits the quality of the biological interpretations that can be drawn from experimental results. In contrast, small molecule drugs may have undefined or incompletely defined modes of action. However, these may be tolerable as long as the agents have appropriate pharmaceutical properties that allow their safe and effective use in humans.

In principle, using kinase inhibitors as chemical probes has a number of advantages over non-pharmacological approaches. For example, methods like gene deletion, RNA interference (RNAi), or expression of mutated proteins require that cells can be efficiently transfected, and also involve long periods of days of weeks. This can allow time for compensatory changes in signaling networks to occur (Shogren-Knaak et al., 2001; Bodenmiller et al., 2010), and can preclude analysis of the distinct functions of a kinase at different stages within a biological process (e.g., the cell cycle) (Moffat et al., 2006). For example, a complete gene knockout prohibits simple analysis of a protein’s role at a late stage of a process if the deletion causes a defect in an earlier step. These methods also do not allow inhibition to be rapidly reversed, so it is hard to study the effect of kinase reactivation after an experimental manipulation. In addition, kinase proteins may have functions unrelated to their enzymatic activity, such as the formation of protein-protein interactions (PPIs). Methods such as RNAi, genetic knockout, or induced protein degradation, therefore may cause alterations in signaling pathways that are not due to altered kinase activity (Weiss et al., 2007; Knight et al., 2010). In contrast, small molecule inhibitors typically act within minutes, they can be added to cells at different times during a cellular assay to test their effect on a particular stage without affecting a preceding one, and they can selectively target the enzymatic activity of the kinase. Inhibitors also can be applied to almost any cell type or cell extract, may allow essentially complete yet reversible inhibition, and can often be used in whole organism studies.

As with any experimental approach, chemical inhibitors also have potential disadvantages. There are over 500 kinases encoded by the human genome (Manning et al., 2002; Wilson et al., 2018), and one issue is the lack of effective inhibitors for many kinases, particularly the understudied proteins of the so-called “dark kinome.” Efforts such as the “Illuminating the Druggable Genome (IDG)” program and the Structural Genomics Consortium are working to fill these gaps and to identify useful inhibitors for all human kinases (Oprea et al., 2018; Wells et al., 2021).

The major pitfall of inhibitor approaches, however, can be summarized very simply: lack of selectivity. This is a huge concern for kinase inhibitors, most of which target the ATP binding site, a region that has some level of structural similarity in all members of the eukaryotic protein kinase family (Cohen, 2002). Seminal studies of a number of well-known “selective” inhibitors on a panel of kinases revealed an alarming degree of promiscuity (Davies et al., 2000; Bain et al., 2007; 2003). Unfortunately, there are numerous examples of publications reporting the application of chemical probes as “selective” inhibitors of target kinases, when there is strong evidence of off-target activity (Arrowsmith et al., 2015). While reasonable specificity of several kinase inhibitors (e.g., Lapatinib) has been confirmed, the ease with which the effects of inhibitors on large panels of kinases can now be tested has revealed that essentially no kinase inhibitor can be considered truly selective for a single kinase (Fedorov et al., 2007; Bamborough et al., 2008; Karaman et al., 2008; Anastassiadis et al., 2011; Davis et al., 2011; Metz et al., 2011; Gao et al., 2013; Elkins et al., 2016).

Does this non-selectivity mean that kinase inhibitors are not useful as tools to understand signaling networks? We believe there is still plenty of opportunity to effectively apply chemical probes in such studies, but that the design of these experiments must acknowledge the inherent limitations of kinase inhibitors. For example, the effects of an inhibitor of a particular kinase can be compared with the effects of additional “orthogonal” inhibitors with different off-target profiles (Davies et al., 2000; Bamborough et al., 2008). Alternatively, as discussed below, datasets can be analyzed in a way that explicitly accounts for the known off-target effects of the probes, or pharmacological methods can be combined with genetic manipulation in chemo-genetic approaches that aim for the “best of both worlds,” for example, where inhibitor selectivity is ensured by making specific mutations in the target kinase (Davies et al., 2000; Shogren-Knaak et al., 2001).

Regardless of these factors, a number of kinase inhibitors have been successful in the clinic for both cancer and non-malignant diseases (e.g., imatinib, gefitinib, tofacitinib and others), and there is an undeniable need to develop additional therapeutic agents that target kinase activity (Cohen et al., 2021). Consequently, it is vital that we develop methods to characterize the effects of such agents on cellular signaling networks. Indeed, the complexity, redundancy, and flexibility of kinase signaling networks within cells are becoming increasingly obvious, and with this comes the realization that we may need to block multiple elements within a signaling network to develop effective treatment options. Polypharmacology refers to the idea that a single agent may be efficacious as a drug because it engages more than one target to cause the desired changes in cell function (Hopkins, 2008; Knight et al., 2010). In this case, the ability of a chemical entity to inhibit more than one kinase can be a benefit rather than a flaw. Clearly, we need “target deconvolution” approaches that can be used to understand how kinase inhibitor drugs are altering kinase signaling networks. In addition, to rationally design poly-pharmacological drugs or combinations of drugs, we must understand the larger signaling network so that we can identify potential combinations of kinases that can be simultaneously targeted for the treatment of specific diseases.

Here, we divide the process of understanding kinase networks using pharmacological approaches into three elements. First, detailed selectivity information on chemical probes must be obtained. Second, individual KSRs within the network must be identified and, third, these KSRs must be integrated (together with additional information) to understand network structure and behavior. We will discuss these three elements in turn.

## 3 Methods for profiling target engagement by kinase inhibitors

For pharmacological approaches to be useful to elucidate kinase networks, the direct targets of chemical probes need to be well-characterized. That is, the molecular entities in cells whose biological function is altered by direct binding to kinase inhibitors must be defined. Acknowledgement of the kinase inhibitor selectivity problem has led to the widespread commercial availability of kinase inhibitor profiling platforms and services. Using these, kinase inhibitors can be rapidly tested for selectivity. In some notable cases, knowledge of “off-target” activity of clinical agents has broadened their clinical utility. This is seen, for example, in the use of imatinib, first developed as an ABL inhibitor for chronic myelogenous leukemia, to target KIT and PDGFR in gastrointestinal stromal tumors (Demetri et al., 2002). The great majority of profiling methodologies test the activity of inhibitors on recombinant kinases *in vitro*. However, native kinases in their cellular environments have different properties that are likely to change their sensitivity to inhibitor action. Because of this, there have been increasing efforts to develop ways to assay inhibitor activity in cell extracts and living cells.

### 3.1 *In vitro* kinase inhibitor profiling to determine target engagement

There are many approaches to measure the activity of recombinant kinases that can be used for characterizing the effects of inhibitor compounds *in vitro*. Broadly speaking, such assays can be divided into those that measure the influence of inhibitor compounds on enzymatic activity, and those that measure the binding of small molecules to kinase proteins (either directly or in competition assays).

#### 3.1.1 Kinase activity assays

Kinase activity assays are beneficial for characterization of chemical probes because they directly measure the property of the enzyme that is the target of inhibitors and of most kinase-directed drugs (*i.e.*, the ability to catalyze a substrate phosphorylation event). In addition, they can provide enzymological information that is valuable for compound optimization and utilization.

Perhaps the “gold standard” format remains the radioactive incorporation assay, typically using [γ-^32^P]ATP or [γ-^33^P]ATP. These assays are robust and sensitive, but do require the handling and disposal of radioactive material (Hastie et al., 2006). A number of providers offer inhibitor profiling assays covering hundreds of human protein kinases in this format, including MRC PPU Reagents and Services, Reaction Biology, and Eurofins. Studies that have profiled large panels of kinase inhibitors by radioactive ATP incorporation assays provide indispensable information for the selection of chemical probes and interpreting their cellular activities (Davies et al., 2000; Bain et al., 2007; 2003; Anastassiadis et al., 2011; Metz et al., 2011; Gao et al., 2013).

Alternative assay formats are available that measure phosphorylation using phospho-specific substrate antibodies, or by changes in substrate peptide charge and/or mass (e.g., mobility shift or IMAP assays from Nanosyn and Carna) or cleavability (e.g., Z-Lyte assays from ThermoFisher), or the generation of ADP (e.g., Adapta assays from ThermoFisher). Such assays are amenable to high throughput profiling, although the use of indirect detection in many of these technologies introduces additional potential for compound interference (e.g., fluorescent inhibitors may interfere with assays that use fluorescent substrates). Again, however, large inhibitor profiling efforts using these methods provide excellent sources of information about the selectivity of numerous inhibitors (Metz et al., 2011; Elkins et al., 2016).

It is worth remembering that essentially all of these profiling approaches use a single peptide substrate for each kinase. In reality, many kinases have multiple substrates and, because the kinetic properties of kinases may be affected by these substrates, it is possible that inhibitor profiles will be different for different kinase substrates (Sommese and Sivaramakrishnan, 2016). Perhaps more importantly, different kinase assay formats use different concentrations of ATP (typically either a fixed concentration such as 1 mM, or a concentration near the K_m_ of each kinase), which influences the inhibition observed for ATP-competitive inhibitors and therefore comparisons between kinases (Knight and Shokat, 2005).

#### 3.1.2 Direct and indirect inhibitor binding assays

An alternative approach to characterizing inhibitor activity is to measure inhibitor binding to target kinases. In general, binding affinity of compounds that are known to be kinase inhibitors has been found to correlate well with ability to inhibit kinase activity (Sutherland et al., 2013; Elkins et al., 2016) but, of course, a small molecule may bind to a kinase without influencing its enzymatic activity. On the other hand, such assays can have the advantage of being able to determine substrate specificities of inhibitors for partially purified kinases as well as inactive kinases, which provides useful insights into kinase kinetics relevant to characterizing inhibitors (Wang and Ma, 2015).

**FIGURE 1:**
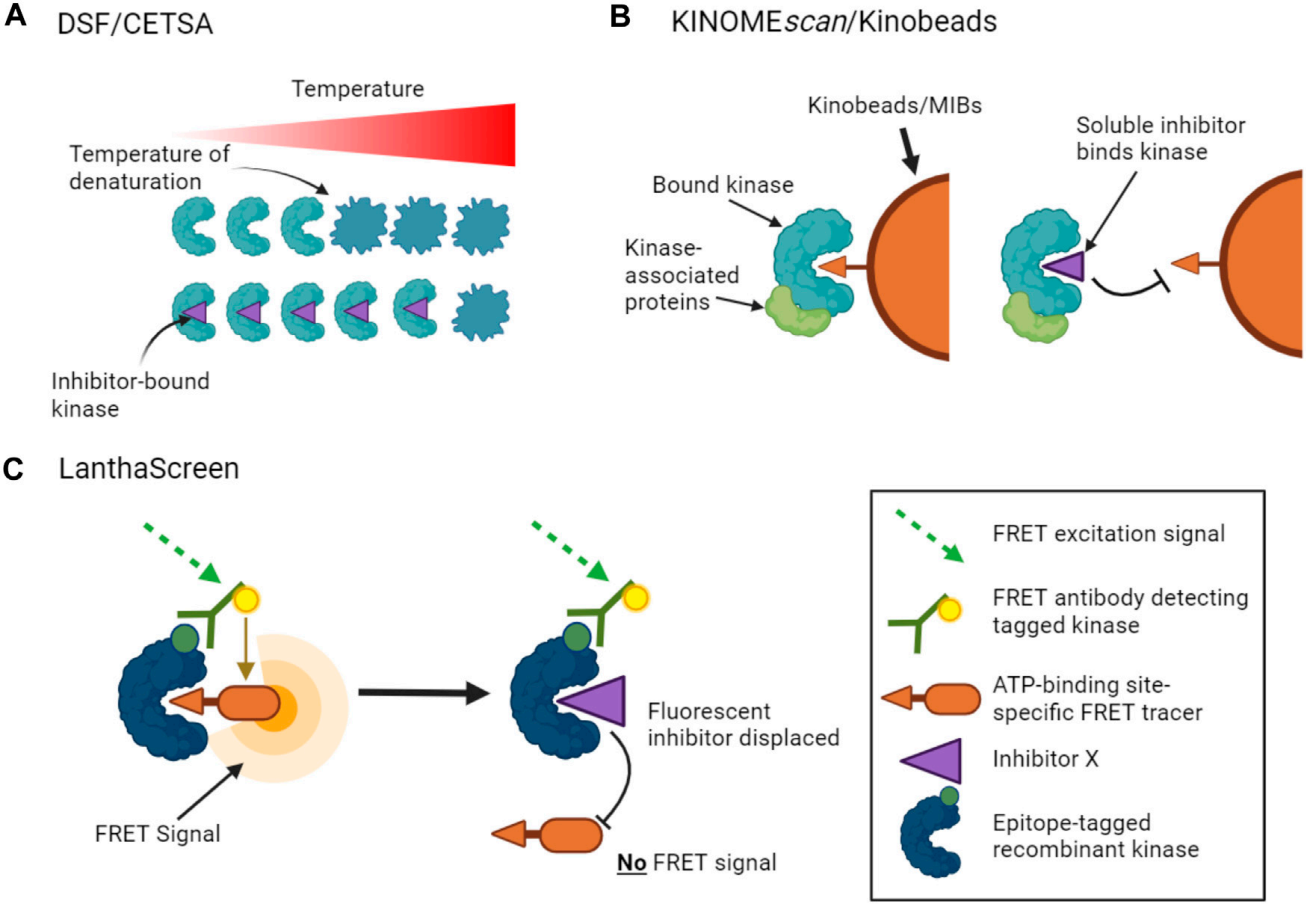
Binding assays to profile target engagement by kinase inhibitors *in vitro*. **(A)**. DSF and CETSA assays infer inhibitor binding by detecting changes in the thermal stability of a kinase upon inhibitor binding. **(B)**. Multiplex inhibitor beads (MIBs) such as Kinobeads display a suite of broad-spectrum kinase inhibitors that can bind recombinant kinases (KINOME*scan* assays) or kinases in cell lysates. Addition of a soluble selective kinase inhibitor displaces only specific kinases from the beads, allowing these kinases to be identified as targets of the added inhibitor. A variant of the assay, kiCCA, measures the displacement of kinase-associated proteins to characterize protein complexes. **(C)**. LanthaScreen binding assays measure target engagement by inhibitors from the reduction of FRET signal when a fluorescent ATP-competitive tracer is displaced from a tagged kinase bound to a fluorophore-labelled antibody.

Thermal stability shift assays provide one method to measure inhibitor binding with the advantage that no additional kinase or inhibitor-specific probes are required. In this approach, the change in stability of a kinase caused by inhibitor binding is measured as the kinase is denatured by heating. There are a number of ways to measure such protein unfolding, but differential scanning fluorimetry (DSF) has been used successfully for kinase inhibitor profiling *in vitro*, in which the increased binding of a hydrophobic dye to denaturing protein is measured (Figure 1A) (Fedorov et al., 2007; Echalier et al., 2014; Elkins et al., 2016). Notably, the method does not distinguish between compounds that bind to the active site *versus* other regions of the kinase.

Other approaches use competition assays to focus attention on the ATP binding sites of kinases. For example, KINOME*scan* assays (from Eurofins/DiscoverX) monitor the capture of tagged recombinant kinases by beads carrying a suite of broad-spectrum kinase ATP-competitive inhibitors (Fabian et al., 2005). The binding affinities of added soluble kinase inhibitors that prevent the interaction of kinases with the immobilized ligands can then be inferred from the reduction in kinase capture by the beads (Figure 1B). KINOME*scan* assays have been used to profile the activities of large panels of ATP competitive inhibitors on hundreds of kinases (Bamborough et al., 2008; Karaman et al., 2008; Davis et al., 2011; Jacoby et al., 2015). LanthaScreen Eu kinase binding assays (ThermoFisher) work on a similar principle, in which the displacement of fluorescent ATP-competitive inhibitors from tagged recombinant kinases is measured by time-resolved FRET (Figure 1C). Inhibitor competition can also be measured in complementation assays including split luciferase experiments (Jester et al., 2012). Such assays can provide information on both ATP-competitive and allosteric inhibitors that reduce binding at the ATP site. Notably, however, these competition binding assays cannot be used to profile inhibitors whose activity is driven by alternative mechanisms such as blocking protein substrate binding.

In summary, a number of well-validated profiling assay formats are available that allow the characterization of kinase inhibitors *in vitro*. These unambiguously provide information about the inhibition or binding of inhibitors to purified kinases. However, even the largest kinase panels currently include only about 400 of the approximately 530 wild type human protein kinases. Because the targets of inhibitors are difficult to predict based on the sequence-similarity of kinases (Bamborough et al., 2008; Anastassiadis et al., 2011), this leaves significant gaps in current *in vitro* profiling data. Furthermore, these assays typically use purified recombinant kinases that will not always replicate the properties of native kinases in cells, which may also be different in different cell types. Finally, these techniques tend to be largely “blind” to the possible effects of small molecules on non-kinase targets. Usually, what researchers really want to know is which targets are engaged and functionally altered *in vivo*.

### 3.2 Kinase inhibitor profiling to determine target engagement in cells and cell extracts

The need to understand target engagement *in vivo* has driven the development of methods to profile inhibitor activity in cell lysates and in living cells. A key problem for these approaches is how to unequivocally identify specific kinases within a complex environment. This challenge has often been met by employing mass spectrometry to characterize kinase targets. Such “chemo-proteomic” approaches bring along their own possible disadvantages, such as a reduction in standardization and throughput compared to *in vitro* profiling, but their potential value cannot be questioned.

#### 3.2.1 Affinity-based profiling

In principle, one way to identify the cellular targets of a chemical probe is to use the compound as an immobilized bait to fish for cellular proteins, and then to identify the captured proteins using mass spectrometry (Daub, 2005). This has the advantage that few assumptions are made about the nature of the proteins bound by the probe, and unexpected non-kinase targets of kinase inhibitors may be found. However, the compound must be derivatized to enable it to be immobilized, and this is likely to hinder binding to some cellular targets. In addition, the method is biased towards more abundant proteins in cells, and it is hard to quantify the affinity of binding interactions (Bantscheff et al., 2007). An adaptation of the method to allow quantification is to measure the ability of “free” (non-immobilized) compounds to compete for binding to the immobilized compound (Sharma et al., 2009). This approach, however, still requires the bespoke synthesis of an immobilized probe for each new inhibitor.

A major step forward in this area was the realization that immobilized broad-spectrum kinase inhibitors could be used to simultaneously capture multiple kinases from cell lysates, as utilized in the *in vitro* KINOME*scan* approach described above (Fabian et al., 2005; Bantscheff et al., 2007; Daub et al., 2008; Sharma et al., 2009). The ability of a free kinase inhibitor to compete for binding to the matrix can be measured by quantitative mass spectrometry, providing a reasonably standardized chemo-proteomic approach for profiling multiple unmodified inhibitors (Bantscheff et al., 2007; Sharma et al., 2009). A good example is known as the Kinobeads approach, in which a selection of broad-spectrum kinase ATP-competitive inhibitors are immobilized on beads (also known as multiplexed inhibitor beads, MIBs, see Figure 1B) (Bantscheff et al., 2007; Reinecke et al., 2019). Optimized Kinobead protocols can profile up to 350 kinases (Reinecke et al., 2019). This approach has several advantages: it does not require labeling of inhibitors or kinases, it can identify a subset of possible non-kinase targets of kinase inhibitors, and it can be applied to a wide variety of cell and tissue lysates from various species. For example, Klaeger et al. applied Kinobeads to evaluate the target spectrum of 243 clinically relevant kinase-targeted drugs in human cancer cells (Klaeger et al., 2017), and to identify ferrochelatase as an off-target of a number of clinically relevant kinase inhibitors (Klaeger et al., 2016). Because proteins identified by this chemo-proteomic approach may bind directly or indirectly to Kinobeads (for example, as protein complexes), follow-up work is needed to distinguish direct from indirect drug targets.

Because ligands are immobilized on beads, Kinobead methods can only be used to capture kinases from cell lysates, and not from intact cells. In an alternative format of such assays, living cells are treated with inhibitors, followed by cell lysis and chemo-proteomic profiling. Assuming many kinase inhibitors have slow off-rates, this might allow the binding of inhibitors to kinases in their truly native state to be assessed. Indeed, differences between pre-lysis and post-lysis inhibition profiles have been observed, for example, for imatinib binding to KIT (Bantscheff et al., 2007). Nevertheless, the potential for confounding pre-lysis effects such as changes in kinase abundance, or post-lysis changes such as loss of cell compartmentalization, mean that these affinity-based approaches do not provide true intracellular profiling.

#### 3.2.2 Activity-based profiling

**FIGURE 2:**
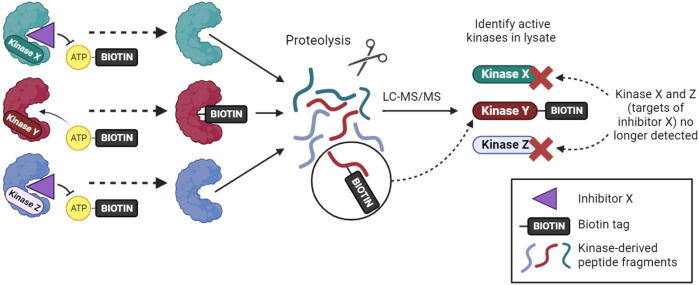
The KiNativ approach to kinase inhibitor profiling. ATP-biotin can bind to kinases within cell lysates, and kinase activity then transfers biotin to a lysine residue in the active site. Addition of a soluble inhibitor can prevent ATP-biotin binding its target kinases, allowing the selectivity profile of the inhibitor to be determined by phosphoproteomic analysis.

An alternative approach for inhibitor profiling in cell lysates is KiNativ. Overall, the method has similarities to Kinobeads, but it uses crosslinking to capture kinases rather than non-covalent affinity interactions. Specifically, when a biotinylated acyl-phosphate derivative of ADP or ATP binds to a kinase (or other ATP phosphohydrolase), the terminal acyl-phosphate is transferred covalently to one of the conserved lysine residues in the active site and the enzyme is thereby tagged with biotin (Figure 2). After limited proteolysis, biotin-containing peptides can be analyzed by targeted mass spectrometry to identify approximately 200 kinases from a single experiment (Patricelli et al., 2011; Patricelli et al., 2007). If capture is carried out in the presence of kinase inhibitors that reduce ATP binding, then KiNativ can be used to characterize inhibitor properties. The technique can thus be considered an activity-based profiling approach that probes the properties of endogenous kinases, and it has been used successfully to profile a number of kinase inhibitors (Patricelli et al., 2011). Despite its name, however, KiNativ remains a cell lysate-based approach and so, arguably, kinases are not in their truly native cellular environment when analyzed.

More recently, cell permeable covalent sulfonyl fluoride ATP binding site probes have been designed (Zhao et al., 2017), and future development may allow broad activity-based kinase profiling in cells. However, not all kinases may be detectable using these methods, perhaps due to their low abundance or lack of suitably placed lysine residues, and the use of covalent binders prevents simple determination of true affinities.

#### 3.2.3 Thermal shift assays in cells

In the quest to develop direct methods for monitoring the binding of drugs to their targets within cells, Martinez Molina et al. tested whether thermal shift assays (see Section 3.1.2; Figure 1A) could be carried out using cells (Molina et al., 2013). Indeed, they found that kinase inhibitors could protect target kinases within cells from denaturation. In this case, kinase unfolding was monitored (using antibodies) as a reduction in the amount of soluble kinase due to heat-induced aggregation and precipitation. The method was named the Cellular Thermal Shift Assay (CETSA). Savitski et al. (2014) combined the approach with mass spectrometry to detect inhibitor binding to over 7000 different proteins including 175 kinases, significantly enhancing the scope for identification of off-target binding compared to other methods. Although it does not yield straightforward K_d_ values, it is useful for comparing the binding of related inhibitors to particular targets. Also, because it is not a direct binding assay, the potential for indirect effects must be noted. For example, changes in post-translational modifications of downstream targets of inhibited kinases can influence their melting temperatures, potentially yielding “false positives.” Indeed, such effects appear more prominent in cellular *versus* lysate thermal profiling experiments (Shi et al., 2014). False negatives have also been reported (Savitski et al., 2014). Nevertheless, thermal proteome profiling (TPP) has the clear advantage that it provides information on kinase and non-kinase target engagement within cells. In one example, ferrochelatase was identified as an off-target of the BRAF inhibitor vemurafenib responsible for kidney toxicity (Savitski et al., 2014; Bai et al., 2021).

#### 3.2.4 NanoBRET

**FIGURE 3:**
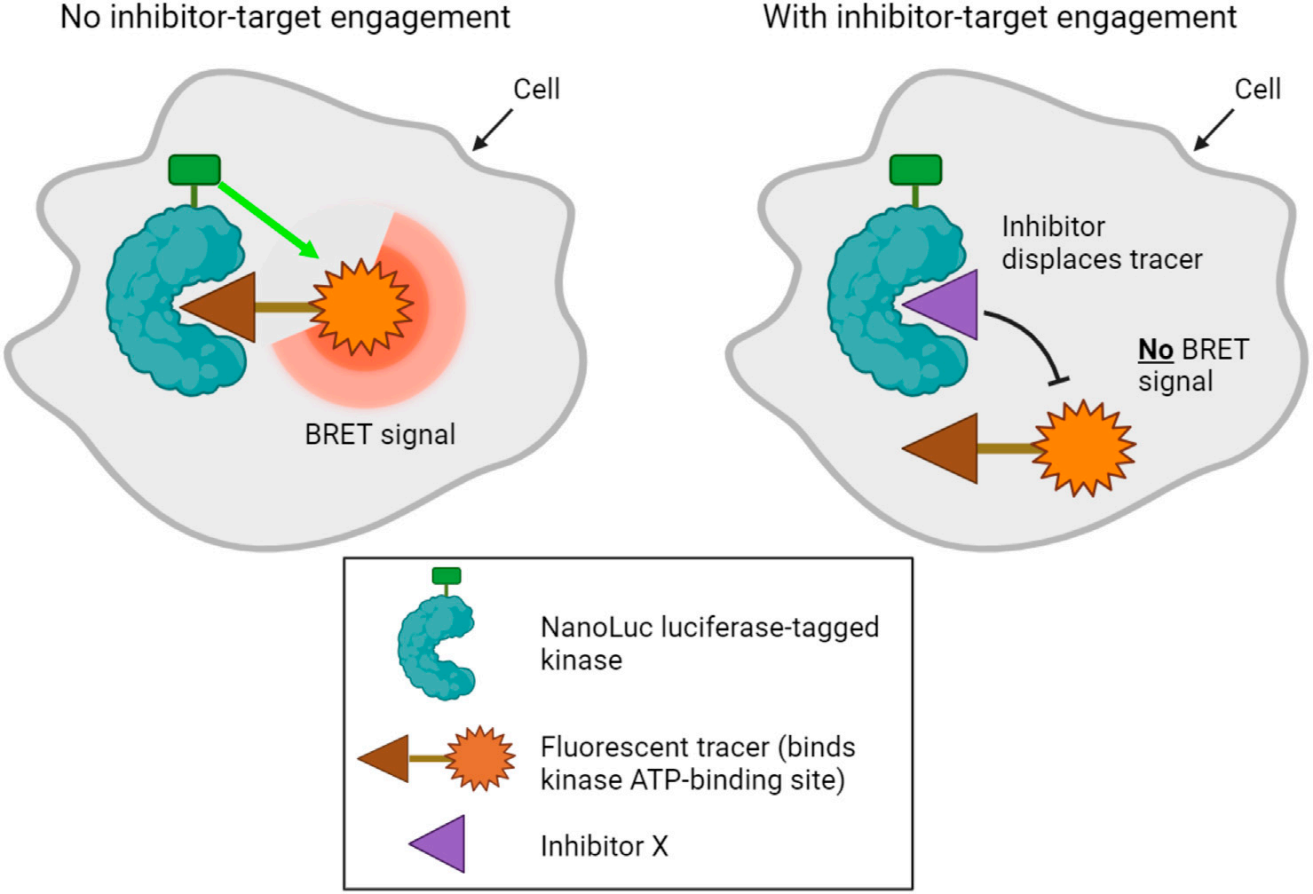
NanoBRET technology used to profile kinase inhibitor target engagement in cells. A bioluminescence signal is emitted when a fluorescent tracer binds the ATP binding site of a luciferase-tagged kinase within cells. Addition of a compound that competitively inhibits the kinase displaces the tracer, disrupting the bioluminescence signal, which can be measured in live cells.

A final method designed to determine kinase target engagement in living cells is NanoBRET technology (Robers et al., 2015; Vasta et al., 2018). This is a bioluminescence resonance energy transfer (BRET) technique that measures the affinity of inhibitors for cellular kinases by competitive displacement of a luminescent tracer from fused NanoLuc luciferase-kinase proteins (Figure 3). Currently, the method only assays one type of kinase per cell, and transfected cell lines that express each NanoLuc-tagged kinase of interest must be made. Furthermore, although NanoLuc is a relatively small protein with a number of advantageous features (Hall et al., 2012), fusion to NanoLuc has the potential to alter the properties of a kinase. Nevertheless, NanoBRET allows the analysis of inhibitor binding to kinases in genuinely living cells.

Vasta et al. (2018) showed that NanoBRET can be used to measure inhibitor selectivity within intact cells for 178 full-length kinases, and Wells et al. successfully profiled 46 cyclin-dependent kinase (CDK) inhibitors on a panel of CDK kinases (Wells et al., 2020). Comparing the results of a NanoBRET assay with other methods revealed important differences in cellular kinase occupancy compared to the results of *in vitro* assays, and suggested that NanoBRET results are more comparable to other cellular assays such as phospho-substrate ELISAs. Importantly for the further development and use of this technique, the activity of the NanoLuc enzyme itself appears to be unaffected by the great majority of kinase inhibitors (Hall et al., 2012; Cartwright et al., 2022). Most current cellular NanoBRET assays make use of overexpressed kinases, but it is possible to use weak promoters or, in future, to tag kinases at their endogenous loci (Vasta et al., 2018; Yang et al., 2023). Efforts to rapidly expand the panel of NanoLuc-fused kinases (Yang et al., 2023) mean that this assay format seems likely to find increased use in the profiling of target engagement in living cells.

In summary, a number of methods have been developed that move researchers nearer to the ultimate goal of quantifying inhibition of kinase function in the native cell environment. Each of the methods has strengths and weaknesses, but NanoBRET technology arguably comes the closest to measuring kinase occupancy in true living cells. While many *in vitro* kinase activity and binding assays have been validated by cross-comparison between the different methods (Sutherland et al., 2013; Tang et al., 2014), broad validation of cell-based methods will need to wait for more widespread adoption of the approaches. It is also worth pointing out that a number of these techniques remain relatively poor at detecting off-target effects on non-kinase targets. Nevertheless, methods such as NanoBRET have great potential to increase our quantitative understanding of kinase inhibitor action *in vivo*.

### 3.3 *In silico* approaches

The availability of large datasets of kinase inhibitor profiles raises the prospect of using *in silico* techniques to predict the target selectivity of kinase inhibitors (Tang, 2017). For example, machine-learning methods have been employed to predict *in vitro* kinase inhibitory activity for large compound libraries (Merget et al., 2017; Li et al., 2019). As experimental datasets grow, machine learning approaches improve, and these methods are extended to cellular inhibition profiling, their utility is likely to increase. Indeed, artificial intelligence is already a key tool in the drug discovery pipeline (Vamathevan et al., 2019).

## 4 Pharmacological approaches to define kinase-substrate relationships

A crucial part of understanding kinase networks is knowledge of individual kinase-substrate relationships (KSRs). Indeed, accurate definition of KSRs is crucial to correctly identify kinase pathways from phosphoproteomic data (Hernandez-Armenta et al., 2017). For this task, pharmacological approaches using well-characterized chemical probes are vital components of the kinase researcher’s toolbox. Note that here we define a KSR as the relationship between a kinase and a downstream substrate it directly phosphorylates. Ideally, “substrate” refers to a single phosphosite within a target protein, but not all methods for identifying KSRs are phosphosite-specific.

### 4.1 Non-pharmacological approaches

There are numerous non-pharmacological ways to characterize KSRs (Johnson and Hunter, 2005). With a cellular assay for phosphorylation in hand (often using a phospho-specific antibody), approaches such as RNAi, genetic knockout and overexpression of kinases can all be used to investigate KSRs. These have limited off-target effects compared to small molecule inhibitors. However, because kinases operate in networks, these methods often identify pathways of kinases that are indirectly responsible for phosphorylation events (Bodenmiller et al., 2010), so other techniques are required to confirm direct phosphorylation events. Furthermore, as mentioned in Section 2, these approaches alter the amount of protein kinases in cells, rather than only preventing catalytic activity, and this may cause additional indirect effects (Weiss et al., 2007).

Biochemical methods such as *in vitro* kinase assays using panels of purified kinases or potential substrates can be used to identify possible KSRs (Ptacek et al., 2005; Newman et al., 2013), but such libraries are usually incomplete and expensive, and are unlikely to fully reflect the activity of kinases in their natural cellular context. Alternatively, in KESTREL and related approaches, purified kinases can be used to seek substrates in cell extracts that are then identified by mass spectrometry (Knebel et al., 2001; Huang et al., 2007; Müller et al., 2016), but these methods are biased towards identification of abundant cellular proteins. Classical techniques in which a specific kinase activity is tracked through biochemical enrichment steps are also possible, but these need large quantities of cells and multiple steps, as well as protein identification techniques to pinpoint candidate kinases (Rubin and Rosen, 1975; Kubota et al., 2009).

For many kinases, particularly serine/threonine kinases, optimal linear phosphorylation motifs have been determined using oriented synthetic peptide libraries or phosphoproteomic analysis of cellular proteins phosphorylated *in vitro* (Songyang et al., 1994; Yaffe et al., 2001; Douglass et al., 2012; Kettenbach et al., 2012; Knight et al., 2012; Xue et al., 2012; Johnson et al., 2023). Specific phosphosites can be compared to these optimal motifs to infer KSRs *in silico*. However, such motifs are not known for all human kinases and are validated for far fewer. Also, natural kinase phosphosites diverge from optimal motifs, because it is likely that sub-optimal regulable phosphorylation is required in signaling networks, rather than phosphorylation at maximal efficiency. In addition, not all kinases may recognize linear motifs, and some kinases certainly make use of binding sites distant from phosphorylation sites to select their substrates (Miller and Turk, 2018). Finally, *in silico* approaches typically lack contextual information such as which kinases are expressed or activated in a specific cellular environment, or the presence of binding partners which may regulate kinase activity. Indeed, it is not necessarily the case that the kinase responsible for phosphorylation of a particular site is the same in different cell types, a point well-illustrated by the rewiring of kinase pathways that can occur in cancer (Knight et al., 2010; Cohen et al., 2021). It is therefore not surprising that false positives and negatives are frequent using such prediction techniques, even when attempts are made to combine them with contextual information such as co-expression or PPI data (Linding et al., 2007).

In sum, it is clear that genetic, biochemical and *in silico* approaches can all be employed to identify KSRs, though they each have limitations. In the rest of Section 4, we explore how the use of pharmacological tools provides an attractive category of complementary approaches.

### 4.2 From kinase to substrate: pharmacological approaches

A number of pharmacological approaches have been developed to determine the target substrates of a specific kinase, most notably phosphoproteomic analysis following inhibitor treatment, and use of chemo-genetic approaches involving modified kinase alleles.

#### 4.2.1 Phosphoproteomics following inhibitor treatment

**FIGURE 4:**
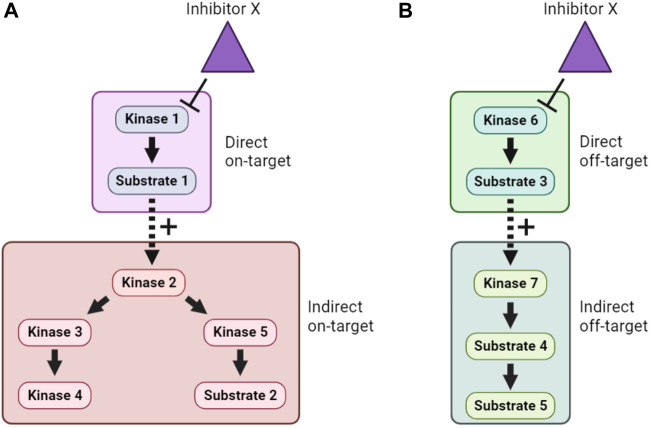
Direct, indirect and off-target effects of kinase inhibitors. **(A)**. In this example, Inhibitor X directly inhibits Kinase 1, and therefore lowers phosphorylation of Substrate 1. However, because Substrate 1 is a positive regulator of Kinase 2, this also causes indirect on-target effects on Kinase 2 and downstream signaling. **(B)**. In addition, if Inhibitor X has direct off-target effects on another kinase, Kinase 6, then both direct and indirect off-target effects on an unrelated cellular signaling pathway can occur. Distinguishing these effects is a major challenge when using inhibitor compounds to study the cellular function of target kinases.

Inhibition of a particular kinase in cells will decrease the phosphorylation of its substrates. Therefore, kinase inhibitor treatment followed by phosphoproteomics has been used to infer KSRs for a number of kinases (Kettenbach et al., 2011; Knight et al., 2012). Even though kinase inhibitors are typically fast-acting, one of the major obstacles to the interpretation of such studies is the need to distinguish direct from indirect substrates. This arises because inhibition of a specific kinase may lead to a cascade of altered phosphorylation in a signaling network, as illustrated in Figure 4A. In many phosphoproteomics studies, identified phosphosites are subsequently compared with optimal sequence motifs defined for the kinase of interest to discriminate direct from indirect substrates. This, however, means that the method suffers from some of the same shortcomings as the *in silico* approaches mentioned in Section 4.1, including that not all *bona fide* substrates conform closely to these motifs. Another major concern is the underlying assumption that only one kinase is inhibited by the probe compound, when it is clear that essentially all kinase inhibitors have additional targets (Figure 4B). Use of more than one distinct kinase inhibitor can mitigate these concerns to some extent, but caution and follow-up experimentation are required.

#### 4.2.2 Chemo-genetic approaches

**FIGURE 5:**
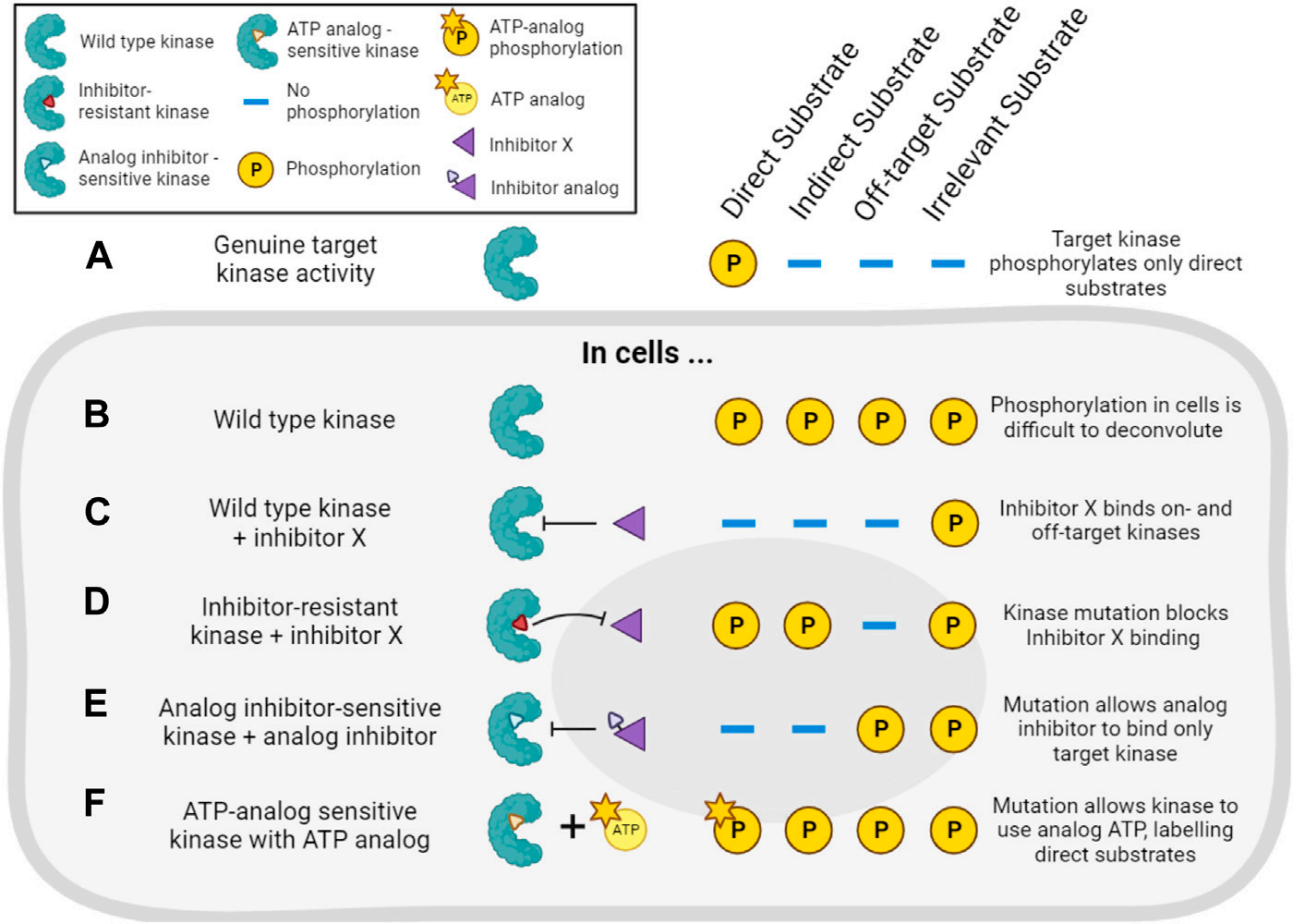
Chemo-genetic approaches for establishing kinase-substrate relationships (KSRs). **(A)**. For the purposes of this figure, we consider a cellular “target kinase” that forms a KSR with a substrate that it directly phosphorylates in cells. By definition, this kinase does not phosphorylate indirect or other potential cellular substrates. **(B)**. In cells, analysis of substrate phosphorylation (for example, by phosphoproteomics) will reveal phosphorylation of the direct substrate, as well as phosphorylation of many other proteins driven by other kinases. **(C)**. Treatment of the cells with a typical incompletely selective kinase inhibitor of the target kinase (“inhibitor X”) will decrease the phosphorylation of direct and indirect downstream substrates, as well as substrates downstream of off-target kinases. **(D)**. In cells expressing an inhibitor-resistant mutant of the target kinase, inhibitor X will no longer decrease phosphorylation of direct or indirect targets of the kinase, but off-target inhibition will still be observed. **(E)**. In cells expressing an analog-sensitive version of the target kinase, treatment with an analog inhibitor will decrease phosphorylation of direct and indirect substrates of the kinase, but will spare the substrates of off-target kinases. **(F)**. In cells expressing a mutated target kinase that can carry out phosphorylation using a bulky ATP-analog, only the direct substrates of the target kinase will be labeled with modified phosphate.

The combination of pharmacological and genetic approaches (“chemo-genetics”) can be used to alleviate concerns about inhibitor specificity (Figure 5). Shokat and co-workers developed an approach in which kinases of interest can be genetically modified to accept bulky ATP-competitive inhibitors that are not able to inhibit the activity of natural protein kinases (Bishop et al., 2001; 1998). By replacing the expression of a wild type kinase with a genetic variant containing a mutated residue in the ATP binding pocket, selective inhibition of only the mutant kinase in cells should be ensured (Figure 5E). Compared with traditional genetic knockouts, the approach still allows acute inhibition of kinase activity using small molecules, reducing the potential for compensatory changes in signaling pathways. These so-called analog-sensitive kinase alleles have undoubtedly been informative, though it remains a challenge to design and express the necessary mutated but active kinases in cells, making this a relatively low-throughput approach. There is also evidence that bulky inhibitor analogs such as 1NM-PP1 have off-target activity on some endogenous kinases such as PKA (Bain et al., 2007; Kanshin et al., 2017), though these off-targets are likely limited and they can be controlled for by examining the effect of the inhibitor analogs on wild type cells.

Analog-sensitive kinases have been used in combination with phosphoproteomics and motif analysis (as described in Section 4.2.1) to identify potential substrates of, for example, Polo kinase-1 (Oppermann et al., 2012). More recently, a sophisticated methodology combining selective chemical inhibition of analog-sensitive kinases, quantitative phosphoproteomics, and machine learning has been reported (Kanshin et al., 2017). Here, the use of bulky inhibitors and modified kinases addressed the issue of inhibitor off-target effects, while a learning classifier aimed to address the common issue of incorrectly identifying phosphorylation events as direct targets of the kinase, when they may really be attributable to downstream effects. The authors used this strategy to identify direct substrates of Cdc28 and Snf1 in budding yeast (Kanshin et al., 2017).

Related chemo-genetic approaches also have potential. For example, kinases can be genetically modified to make them resistant to small molecule inhibition (Figure 5D). This approach is useful to validate that a specific kinase is involved in the phosphorylation of a substrate that is decreased by a particular kinase inhibitor (Davies et al., 2000). Kinases also can be engineered to accept bulky ATP analogs that cannot be utilized by wild type kinases (Figure 5F). In this way, substrates downstream of the kinase of interest can be specifically labeled, and this has been used to identify Cyclin-dependent kinase substrates in cell lysates, for example, (Shah et al., 1997; Allen et al., 2007; Blethrow et al., 2008; Chi et al., 2008; Michowski et al., 2020). However, these approaches require extensive validation that modified kinases are still capable of wild type kinase activity, and not all kinases are amenable to this form of modification. Auxin-Inducible Degradation (AID) is another chemo-genetic approach that might be exploited to acutely remove kinase activity from cells (Nishimura et al., 2009). For most of these methods, distinguishing indirect *versus* direct effects of inhibitors remains the major problem. Nevertheless, pharmacogenetic approaches offer an enticing alternative to genetic knockouts to study a kinase’s activity in live cells.

### 4.3 From substrate to kinase: pharmacological approaches

In addition to pharmacological approaches which aim to identify the substrate of a kinase, approaches have been developed that achieve the reverse, that is to identify the kinase responsible for a given phosphorylation event. These methods are particularly important for understanding the kinases involved in cellular phosphorylation events uncovered in large scale phosphoproteomic studies, for example,.

#### 4.3.1 Cross-linkable ATP analogs

In principle, kinases could be identified based on binding to their substrates, but such interactions are often transient and hard to capture. However, methods have been developed that use modified substrates and/or ATP analogs to covalently cross-link specific substrates to wild type kinases. In one approach, a modified substrate containing a reactive cysteine in place of the phosphorylation site is combined with a cross linkable ATP analog to enable activity-based crosslinking to kinases (Maly et al., 2004; Statsuk et al., 2008). In another, an ATP analog containing a UV-activated reactive group allows crosslinking of kinases to a specific biotinylated substrate peptide (K-CLASP) or endogenous substrate (K-CLIP) (Dedigama-Arachchige and Pflum, 2016; Garre et al., 2018). Complexes containing crosslinked substrate and kinase can be purified by streptavidin pulldown or immunoprecipitation. Predicted KSRs can then be tested using western blots or, alternatively, unknown KSRs can be identified by proteomic analysis (Dedigama-Arachchige and Pflum, 2016; Beltman and Pflum, 2022). Not all kinases can utilize unnatural ATP analogs to phosphorylate substrates and another disadvantage is that, because the ATP analogs do not cross cell membranes, these techniques cannot be performed in cells. They can, however, be carried out using a variety of cell and tissue homogenates. These approaches may also not be effective at identifying kinase interactions for low abundance substrates, and K-CLASP and K-CLIP are prone to crosslinking interacting proteins in addition to the relevant kinase and substrate. On one hand, this lowers the specificity for kinase identification, but on the other hand provides additional information about the context of phosphorylation reactions (Dedigama-Arachchige and Pflum, 2016; Garre et al., 2018).

#### 4.3.2 Kinome-wide inhibitor screens

One approach to identify the kinase responsible for a specific phosphorylation event is to genetically deplete or overexpress all possible kinases and determine if the expected change in phosphorylation occurs (Friedman and Perrimon, 2006; Azorsa et al., 2010). As described above, these are relatively long-term experiments, and the nature of cellular signaling networks make indirect effects common. Methods that use kinase inhibitor libraries with the aim of mimicking one-agent-one-kinase genetic screens allow more acute inhibition of kinases and, with appropriate experimental design, can lower (but not eliminate) the potential for indirect effects. For such approaches to be broadly applicable, libraries with a high coverage of the kinome and the most selective inhibitors possible for each kinase must be assembled, and tools to facilitate design of such libraries have been developed (Drewry et al., 2017; Moret et al., 2019). An example of such an open source library is the kinase chemogenomic set (KCGS) assembled by the Structural Genomics Consortium (Elkins et al., 2016; Wells et al., 2021). KCGSv1.0 contains 187 potent kinase inhibitors that each have a narrow spectrum of activity when screened on a large panel of kinases *in vitro*. These inhibitors cover 215 human kinases using the consortium’s potency and selectivity criteria. More recently this has been superseded by KCGSv2.0 which includes 295 kinase inhibitors with activity on 262 kinases. Nevertheless, few, if any, kinase inhibitors can be considered truly selective for one kinase.

**FIGURE 6:**
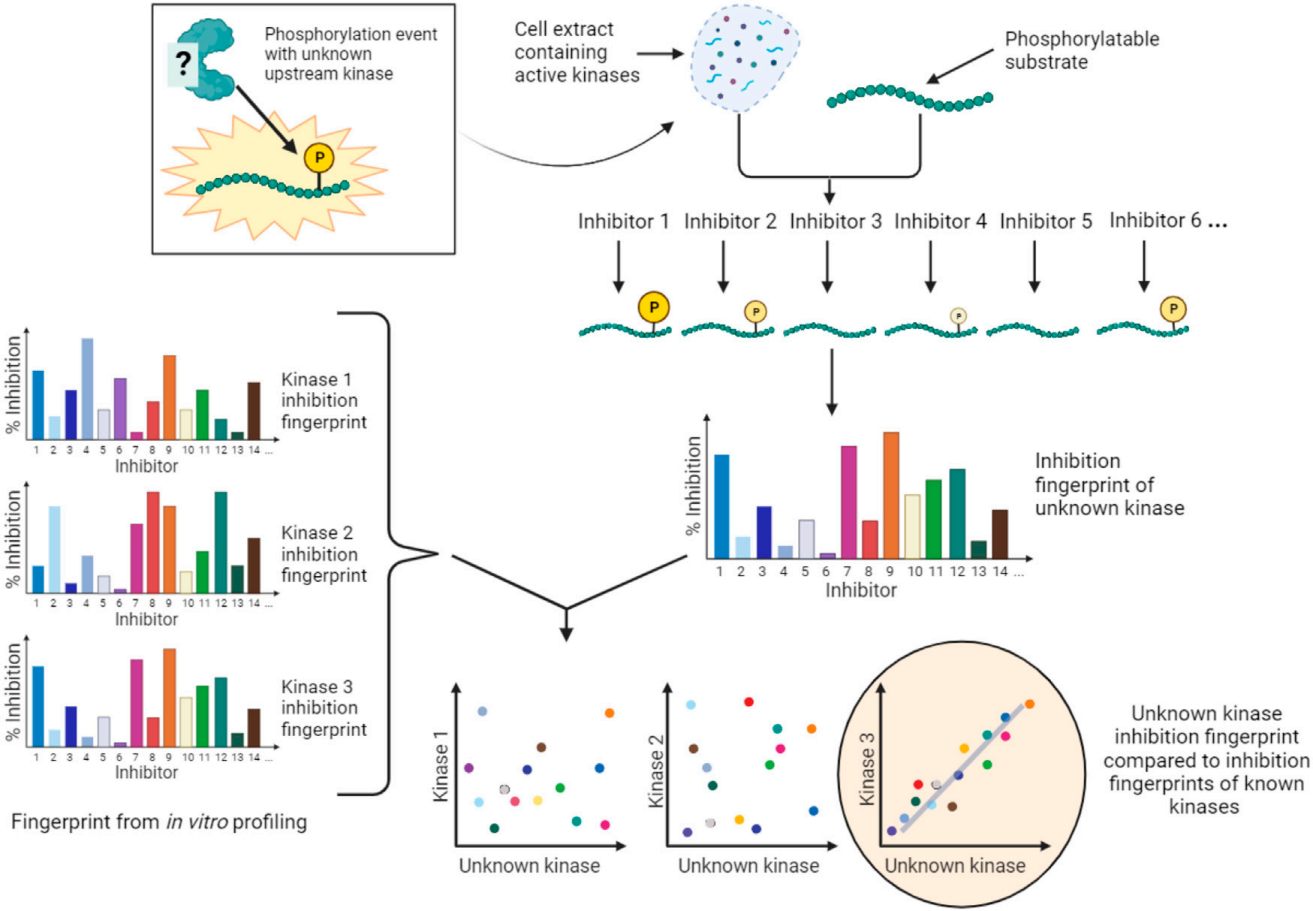
KiPIK assay to identify kinases for unassigned phosphosites. In KiPIK, extracts of cells in which the phosphosite of interest is known to be phosphorylated are used as the source of all relevant kinases. In the presence of ATP, multiple kinase reactions are then carried out in parallel, each in the presence of a single well-characterized kinase inhibitor. This produces an inhibition fingerprint for the unknown kinase that is driving phosphorylation of the substrate added to the cell extract. This fingerprint is then compared (by correlation coefficients) to known inhibition fingerprints derived from *in vitro* inhibitor selectivity data available for the majority of kinases in the human kinome. Kinases are then ranked based on these correlation coefficients to identify kinases most likely to be the direct kinase responsible for the phosphorylation event being studied.

An alternative conceptual approach is to accept that the majority of kinase inhibitors are not selective for a single kinase, and to develop kinase screening methods that exploit the known off-target effects of extensively profiled small molecule inhibitors. An example of this type of approach is Kinase inhibitor Profiling to Identify Kinases (KiPIK; Figure 6) (Watson et al., 2020). In this method, whole cell extracts are used as the source of all potentially relevant kinases to drive phosphorylation of an exogenous substrate of interest. Multiple such cell extract kinase reactions are carried out in parallel, each in the presence of a member of a panel of well-characterized kinase inhibitors. This yields an inhibition fingerprint that characterizes the cellular kinase mainly responsible for the observed phosphorylation. Then, to identify candidate kinases, this fingerprint is compared to the known inhibition fingerprints of all kinases in published profiling datasets (Fedorov et al., 2007; Anastassiadis et al., 2011; Davis et al., 2011; Gao et al., 2013; Elkins et al., 2016), which constitutes approximately 80% of human protein kinases. This step relies on the finding that inhibition profiles of kinase inhibitors in cell extracts are generally similar to those established *in vitro* (Bantscheff et al., 2007; Sharma et al., 2009; Patricelli et al., 2011). The technique has been validated using a number of known KSRs and successfully applied to identify cellular kinases for unassigned phosphosites such as a non-canonical Cdk1 site in the protein INCENP (Watson et al., 2020; Cartwright et al., 2022). KiPIK utilizes the rapid action of kinase inhibitors in cell extracts to focus on defined biological states. Indeed, there is evidence that the use of cell extracts can minimize the influence of upstream kinase signaling (Wang et al., 2011; Savitski et al., 2014; Watson et al., 2020), aiding the identification of direct KSRs, but indirect effects cannot be fully ruled out. KiPIK also rests on the assumption that kinase reactions in cell extracts preserve physiological kinase-phosphosite dependencies which may often, but not always, be true.

In summary, there are a number of pharmacological and chemo-genetic approaches that provide potential advantages over genetic methods for identifying direct KSRs, and advantages over predictive methods for revealing context-specific KSRs, as long as the limitations of kinase inhibitor selectivity are taken into account.

## 5 Pharmacological approaches for analyzing kinase signaling networks

So far, we have discussed approaches to characterize small molecule kinase inhibitors and to identify KSRs. Here, we outline how integrating this knowledge *in silico* (often with additional information) allows insight into the structure of kinase signaling on a network scale. We also discuss how experiments with well-characterized inhibitors can be used to probe signaling networks and to identify kinases that are involved in specific phenotypic responses. No matter how thoroughly a small molecule inhibitor has been characterized against its intended target and the wider kinome, we still need to understand the effects of inhibitors on specific cellular kinase networks, and to be able to predict which patients will respond to treatment. We describe ways in which these factors can be assessed in cell line and patient-specific ways.

### 5.1 Constructing kinase network models

**FIGURE 7:**
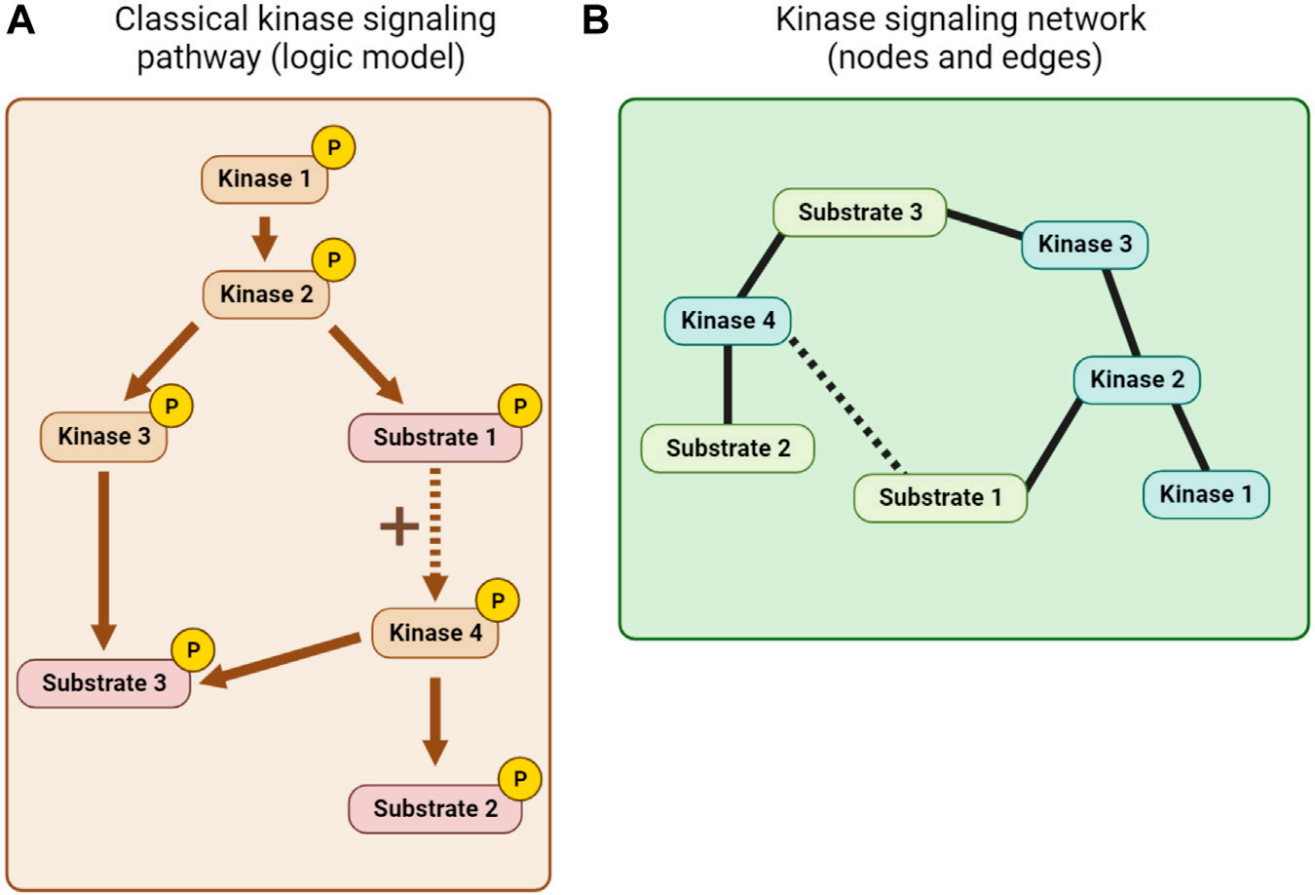
Kinase network visualization. **(A)** A classical kinase signaling pathway or logic model derived from KSR, PPI and other mechanistic information. **(B)** A node and edge kinase signaling network derived, for example, from KSR and PPI information.

Kinase pathway diagrams (see Figure 7A), which are essentially informal logic models, have long been a feature of cell signaling research. Early efforts to integrate information beyond single pathways focused on the use of KSRs to build networks of nodes (kinases and substrates) and edges (interactions between them; see Figure 7B). The KSRs used in these networks were determined by *in vitro* kinase assay screens (Ptacek et al., 2005; Newman et al., 2013) or predicted from optimal motif analysis, sometimes combined with additional information such as PPI or co-expression data (Linding et al., 2007; Song et al., 2012). Alternative kinase network models were also built using genetic or PPI data (Fiedler et al., 2009; Breitkreutz et al., 2010). Inevitably, the quality of such networks is limited by the quality of the experimentally-determined and predicted associations, and the scope of the datasets.

The rise of quantitative phosphoproteomics has allowed the status of kinase signaling to be monitored in unprecedented breadth and in specific cellular contexts. For example, a now classic report examined the spreading of phosphorylation through an EGF-induced signaling network over time in HeLa cells (Olsen et al., 2006). A limitation of phosphoproteomic studies is that they do not themselves delineate connections between kinases and their substrates, so approaches using known KSRs to predict the upstream kinases for phosphosites and to identify up or downregulated signaling pathways are crucial. In addition, the peptide under sampling inherent in current whole cell phosphoproteomic dataset acquisition influences the types of approaches that can be used for network building (Terfve et al., 2015). A number of algorithms have been developed, including those akin to gene set enrichment analysis (GSEA) (Drake et al., 2012; Weidner et al., 2014; Ochoa et al., 2016), parametric Z-tests (KSEA) (Casado et al., 2013; Wiredja et al., 2017), or heuristic machine learning (IKAP) (Mischnik et al., 2016). Benchmarking these approaches against a high-quality set of curated KSRs provides confidence in a number of them, but confirms that the quality of the input KSRs used is paramount (Hernandez-Armenta et al., 2017). Alternatively, the combination of phosphoproteomic data with KSRs allows the construction of logic models that may provide greater mechanistic insight into network function (Vaga et al., 2014; Terfve et al., 2015; Schäfer et al., 2019). Machine learning approaches may also help distinguish direct and indirect substrates (Kanshin et al., 2017). Notably, though, assuming that KSRs are generic, and using KSRs determined in one environment in another, decreases the confidence with which context-specific network features can be uncovered.

As the field has developed, additional information beyond KSRs has been integrated into kinase network models, including PPIs, kinase interaction partners, phosphatases, co-regulation of phosphorylation, genetic mutation, expression data, and structural information (Breitkreutz et al., 2010; Reimand et al., 2013; Yang et al., 2015; Domanova et al., 2016; Drake et al., 2016; Rudolph et al., 2016; Yadav et al., 2017; Ayati et al., 2019; Buljan et al., 2020; Invergo et al., 2020; Bello et al., 2021a; Yılmaz et al., 2021). However, because kinase networks have a high level of plasticity and are partly context-specific, this cannot always be fully uncovered by combining experimental data obtained from different cell lines or tissues. Here, pharmacological approaches to understanding kinase networks are likely to help answer a number of important questions.

### 5.2 Can we improve knowledge of networks using pharmacological methods?

When used appropriately, chemical probes can be used to increase our understanding of signaling network wiring. Many phosphoproteomic studies that aim to model kinase network changes upon kinase inhibitor treatment use KSR information to build network graphs or logic models (Terfve et al., 2015; Wilkes et al., 2015), or other interaction information (such as the STRING database) to produce networks (Bose et al., 2006; Pan et al., 2009; Stuart et al., 2015). Comparison of phosphoproteome changes in response to multiple different perturbations can increase the power of such analyses (Ochoa et al., 2016). Notably, these studies typically analyze data on the assumption that the inhibitors used are selective for particular kinases. As we have seen, this is rarely the case. Usefully, in some cases, two distinct inhibitors were used for each nominal target to provide additional confidence in the results (Terfve et al., 2015; Wilkes et al., 2015).

More recently, an alternative approach was used in which broad selectivity information from the *in vitro* inhibition profiles of a panel of kinase inhibitors were used to identify kinase-phosphosite relationships from proteomics data. In these experiments, quantitative phosphoproteomics was utilized to monitor phosphorylation changes in cells treated with a panel of well-characterized kinase inhibitors. This approach is comparable to the KiPIK method (Section 4.3.2) in that it makes use of the incomplete specificity of kinase inhibitors rather than ignoring it. However, it differs from KiPIK because it uses intact cells, not cell extracts, and it does not seek to identify direct KSRs. Instead, it uses an “expectancy of being downstream target” (EBDT) algorithm to place phosphosites that might be directly or indirectly downstream of a particular kinase into groups of putative downstream targets (PDTs). This information can then be used to create context-specific network models in a way that does not depend on prior knowledge of KSRs or other information, and which can identify possible tumor vulnerabilities, for example, TTK in acute myeloid leukemia (AML) (Hijazi et al., 2020). This is a potentially powerful approach, though it depends on the assumption that kinase inhibitor specificity in living cells is similar to that *in vitro*.

The principle of using inhibitor selectivity profile information has been further exploited in another recent study which aimed to identify protein binding partners of specific kinases. In this work, the ability of a panel of kinase inhibitors to displace kinase-containing protein complexes from Kinobeads (see Figure 1B) was determined. Similar to KiPIK, by correlating the known inhibition fingerprints for kinases with displacement fingerprints of putative kinase-associated proteins determined by mass spectrometry, the “kiCCA” method was able to identify proteins associated with 238 different kinases. In this way, local context-specific kinase PPI networks can be examined (Golkowski et al., 2023).

### 5.3 Which kinases are good targets to bring about a specific change in cell phenotype?

In many cases, there may be a cellular phenotype that we wish to modulate with drugs for therapeutic benefit. This might be as conceptually simple as killing proliferating cancer cells, or more complicated, such as modulating the immunoregulatory functions of a particular cell type. In other situations, we may need to understand how kinases contribute to phenotypes we wish to avoid, such as drug-induced toxicity. In such systems, it is likely that multiple kinases influence the phenotypes in question, and we know that most drugs influence the activity of more than one kinase. Therefore, methods for “target deconvolution” are required that can tell us which kinases are actually involved in phenotypic changes so that we can understand the underlying signaling pathways and identify targets for drug development. Pharmacological approaches to address these questions have been developed that exploit kinase inhibitor selectivity data (see Section 3) in combination with the effects of inhibitors in phenotypic screens. These methods therefore have conceptual similarities to KiPIK, EBDT, and kiCCA (see Sections 4.3.2 and Section 5.2, and Figure 6).

In 2013, Tyner et al. (2013) published a study in which the ability of 66 kinase inhibitors to decrease leukemia cell proliferation was tested. To determine which kinases were responsible for the effect, they used a weighted scoring system based on the known kinome inhibition profiles of the 66 inhibitors to identify and rank the common kinase targets of effective inhibitors. Using this approach, they were able to successfully identify kinases driving proliferation in patients with oncogenic kinase mutations such as in FLT3 or ABL1. Ryall et al. (2015) described a similar Kinase Addiction Ranker (KAR) algorithm which appeared effective in identifying kinases responsible for the drug sensitivity of lung cancer cell lines. In somewhat analogous methods, Pemovska et al. (2013) combined drug sensitivity data with kinase selectivity profiles to provide insight into particular kinases involved in AML, and Sundberg et al. (2014) ranked kinases that suppressed IL-10 production by dendritic cells. Another feature selection algorithm uses kinase inhibition profiles and the response of cells to drugs to infer kinase circuits involved in tumor proliferation (Berlow et al., 2013).

In an interesting variation on this theme, Lamore et al. (2017) wished to identify kinases responsible for the wide-spread problem of drug cardiotoxicity. First, they measured the effect of a panel of well-profiled kinase inhibitors on cardiomyocyte beating, providing a proxy fingerprint for cardiotoxicity. Then, they used a univariate correlation analysis to compare this fingerprint to the inhibition fingerprints of all kinases in the profiling dataset. This provided a ranked list of kinases that might be involved in the cardiotoxicity. In an alternative approach, they also narrowed down “sentinel kinases” with the highest predictive value using a classification tree based on recursive partitioning. Testing of drugs with known clinical cardiac warnings showed that, indeed, the majority inhibited these sentinel kinases (RPS6KB1, FAK, and STK35).

Other studies have used an alternative algorithm for deconvoluting kinases involved in cellular responses to kinase inhibitors: regression and variable selection. Kinome Regularization (KiR), uses elastic net regularization to whittle down the list of possible kinases to identify a minimal set with the most power to explain the inhibition of a phenotype. This approach was used to determine which kinases were involved in the inhibition of cell migration by a panel of well-characterized kinase inhibitors, and these were then validated by additional experiments including RNAi (Gujral et al., 2014a; Gujral et al., 2014b; Rata et al., 2020). KiR has also been used successfully to characterize kinases involved in prostate cancer cell proliferation, and in malaria parasite persistence in hepatocyte cultures (Arang et al., 2017; Bello et al., 2021b). Going a step further, the KiR algorithm can also be used successfully to predict polypharmacological inhibitors or combinations of inhibitors that will induce desired changes in cell behavior, even before the inhibitors have been tested in the phenotypic assay (Gujral et al., 2014b; Bello et al., 2021b). KiR has recently been expanded by integrating prior knowledge of PPIs to uncover kinase-focused cellular PPI networks (Bello et al., 2021a).

Machine learning approaches have also been applied to target deconvolution. Following a screen for neurite outgrowth using a kinase inhibitor library, a maximum relevance and support vector machine algorithm (MR-SVM) was used to exploit kinome profiling data to identify a small number of kinase groups that are most relevant in controlling axon growth. Significant hits could be validated by RNAi, and the results allowed the rational selection of compounds with complementary polypharmacology for further study (Al-Ali et al., 2015).

Notably, a major advantage of all these pharmacology-based deconvolution methods is that they provide context-specific information. Indeed, different kinases were identified as important in different cell lines or patient samples in a number of these studies (Tyner et al., 2013; Gujral et al., 2014b; Ryall et al., 2015). Because of this, these approaches could be applied to the study of other context-specific networks, such as cells that acquire resistance to cancer therapeutics. Further work is needed to fully validate these methods, and to determine which prediction algorithms are most reliable, but the underpinning idea to make use of in-depth inhibitor selectivity data is a powerful one.

### 5.4 How is a kinase network influenced by a drug or chemical probe?

A common research question is how is the signaling network of a particular cell type or tissue influenced by a particular small molecule. For well-characterized chemical or chemo-genetic probes, answering this question can provide information on the direct and indirect targets of the kinase(s) of interest. For drugs, we can learn broadly about their mechanisms of action, including off target effects, similarities to the effects of other drugs, or how inhibitor resistance develops. Clearly, genomic and transcriptomic approaches such as monitoring gene expression changes in response to inhibitors (Subramanian et al., 2017), and the global phosphoproteomics methods discussed above (see Section 5.1 and Section 5.2) have a vital role to play in such studies (Bose et al., 2006; Pan et al., 2009; Stuart et al., 2015; Terfve et al., 2015; Wilkes et al., 2015; Zecha et al., 2023). These approaches can, however, be augmented or complemented by additional methods that are particularly relevant for kinase networks (Pierobon et al., 2015).

For example, to reduce the complexity of phosphoproteome analysis and increase robustness, quantitative measurement of phosphorylation can be focused on a subset of key phosphosites, such as those known to be involved in major cancer signaling pathways. In AQUA, or in “kinase activity assay for kinome profiling” (KAYAK), suites of standard peptides facilitate quantification of phosphorylation by mass spectrometry (Gerber et al., 2003; Kubota et al., 2009), or targeted modes of mass spectrometry can be used (Beck et al., 2017; Payne and Huang, 2017). A number of antibody-based multiplex assays are available that can be used to quantify phosphorylation of specific phosphosites in cell extracts [e.g., MILLIPLEX (Merck) and scioPhospho (Sciomics)]. Such array techniques have been used, in combination with gene expression data, to analyze the consequences of sequential treatment of drugs including kinase inhibitors on the apoptosis of breast cancer cells (Lee et al., 2012). Alternatively, phosphorylation of substrate peptide microarrays such as PamChip can be used to profile kinase activities in lysates of cells or tissues during a variety of treatments (Lemeer et al., 2007; Keersmaecker et al., 2008). These assays include only a fraction of the possible cellular phosphorylation sites, and the particular kinases involved must be inferred based on previously known KSRs. Nevertheless, this system allows rapid comparison of multiple conditions, and works with small samples such as human BRAF-V600E melanoma biopsies (Tahiri et al., 2013).

Because the development of resistance to targeted kinase therapy is so common, approaches to discover the underlying causes are vital. The first mechanisms of resistance to BCR-ABL inhibitors were identified in imatinib-treated CML patients, revealing *BCR-ABL* gene amplification, or point mutations in the ABL kinase domain that prevented drug binding (Gorre et al., 2001). Potential resistance mechanisms can also be identified using inhibitors *in vitro*. For example, isolation of cells that were resistant to killing by imatinib in cell culture uncovered ABL mutation-independent mechanisms, as well as *BCR-ABL* amplification (Mahon et al., 2000). Other studies combined directed mutagenesis of the ABL kinase domain with *in vitro* exposure to imatinib to identify additional kinase domain mutations that reduced inhibitor efficacy (Azam et al., 2003). More recent *in vitro* studies of kinase inhibitor resistance have identified non-genetic mechanisms of resistance such as epigenetic changes in gene expression, and have suggested that there is a wide diversity of such resistance mechanisms, even within genetically homogenous cell including clonal BRAF-V600E melanoma cells (Sharma et al., 2010; Goyal et al., 2023). The growing sophistication of *in vitro* cancer models and the use of animal tumor models is likely to further enhance the clinical relevance of similar studies of kinase inhibitor resistance in future.

### 5.5 Can kinase network features be used as biomarkers of drug efficacy?

Another clinically significant need is to predict patient-specific responses to kinase inhibitors so that appropriate personalized treatment regimens can be designed, both upon first diagnosis and if resistance to therapy subsequently develops. Currently, this often takes the form of specific biomarkers that indicate susceptibility to a particular drug, such as a mutation in the gene for a kinase. In other cases, “functional diagnostic” approaches might be used: for example, assays that test whether patient cells are susceptible to killing with a specific drug or combination of drugs. These functional approaches may uncover vulnerabilities that are not predicted by genetic analysis, and have been used to guide patient treatment, such as in refractory AML (Pemovska et al., 2013; Tyner et al., 2013; Crystal et al., 2014; Friedman et al., 2015; Robers et al., 2015).

Characterization of kinase network features provides another avenue to identify prospective biomarkers. Early studies that linked drug sensitivity to the genetic features of the NCI-60 panel of cell lines have matured and now include hundreds of compounds tested on almost 1000 cancer cell lines (Weinstein et al., 1997; Barretina et al., 2012; Garnett et al., 2012; Iorio et al., 2016). Such work has revealed associations between drug sensitivity and gene expression changes or mutations in kinase pathways, including during the development of drug resistance (Garnett et al., 2012; Pemovska et al., 2013). However, non-genetic changes are also important, and similar association studies can also be carried out using phosphoproteomics with the aim of identifying non-genetic features of kinase signaling that correlate with drug sensitivity (for example, in the EGFR signaling pathway (Guo et al., 2008)). Notably, suitable markers for drug sensitivity may not always be obvious components of the pathway presumed to be targeted by the inhibitors in question (Alcolea et al., 2012; Casado et al., 2013), illustrating the value of broad phosphoproteomic analysis.

In some cases, knowledge of multiple features of a phosphorylation network may provide improved prediction of patient responses compared with a single feature such as mutation of the gene encoding the perceived target kinase. For example, in a pre-clinical study of patient-derived AML cells, Casado et al. (2018) found that a phosphoproteomic signature predicted the *ex vivo* response to the FLT3 kinase inhibitor midostaurin better than FLT3 mutational status. Importantly, kinases that contribute to such newly discovered signatures, or other regulators such as epigenetic modifiers, may also provide new targets for drug discovery (Casado et al., 2018; Pedicona et al., 2022). Drug sensitivity signatures may also be discovered using more targeted approaches such as PamChip (see section 5.4) (Elst et al., 2011) or using Kinobeads to enrich for kinases (Cooper et al., 2013; Golkowski et al., 2023; 2020).

Because of the expected complexity of predictive signatures, there have been numerous efforts to use machine-learning algorithms to integrate pharmacologic and ‘omic data to predict patient outcomes in response to kinase inhibitors and other drugs. Many of these approaches rely on genomic and gene expression information (Basu et al., 2013; Yang et al., 2013), but more recently the utility of (phospho) proteomic data has been explored. For example, drug ranking using machine learning (DRUML), developed by the Cutillas group, learns to rank cancer drug activity in patients based on a training dataset of drug sensitivity data for a panel of cell lines with corresponding proteomic, phosphoproteomic, and RNA-seq datasets (Gerdes et al., 2021).

## 6 Conclusion

As we have seen, chemical and chemo-genetic probes are undoubtedly useful for the discovery of KSRs and characterization of context-specific signaling networks. However, the target profiles of such probes first must be comprehensively characterized. Numerous techniques are now available to achieve this, but they still have some disadvantages. For example, kinase panels for *in vitro* profiling of inhibitors remain incomplete. So-called “dark kinases” are often absent, and coverage of mutant kinases that are involved in disease and drug resistance remains rather haphazard. Broadening these profiling panels is important both for understanding possible off-target effects, and for widening the net of target deconvolution approaches that rely on extensive inhibitor selectivity profiling of multiple kinases (such as KiR, EBDT, KiPIK and kiCCA).

Despite advances in profiling kinase inhibitor target engagement in living cells, such technologies remain relatively low throughput and are often complicated to perform. For *in vitro* assays, radiolabeled ATP assays are usually considered the “gold standard”, but the limited data obtained so far using cell and lysate inhibitor profiling makes it harder to define their limitations and to validate the results of methods such as CETSA and NanoBRET. Nonetheless, understanding target engagement in cells is the ultimate objective of most studies, so developments in this area are keenly anticipated.

Importantly, many profiling methods exclude possible non-kinase targets of inhibitors, or provide incomplete coverage. Such off-target interactions have the potential to dramatically alter the *in vivo* effects of drugs and chemical probes, and more attention to this aspect of inhibitor action would be welcome. Nevertheless, we have never been in a better position to comprehensively determine the target engagement profiles of kinase inhibitors, both *in vitro* and in cells.

It is clearly important to continue growing our understanding of individual kinase-substrate interactions. However, KSRs can be context specific; the kinase that phosphorylates a particular phosphosite may change depending on which upstream signaling pathway is activated, or from cell type to cell type (Sapkota et al., 2001; Zecha et al., 2023). Network models based on generic KSRs will not reflect these particularities. It is therefore imperative that we understand KSRs in the context of specific signaling networks. The ease with which chemical probes can be employed in different situations has significant advantages for such studies.

Developments in computational approaches are also likely to be key to unlocking the potential of pharmacological approaches to understand protein kinase signaling networks. Efforts to rationalize the inhibitor libraries used for phenotypic studies, either to create libraries of high selectivity for each target kinase (Moret et al., 2019; Wells et al., 2021) or suitable for deconvolution approaches (Gujral et al., 2014b; Rata et al., 2020; Watson et al., 2020; Golkowski et al., 2023), will help optimize the trade-off between library size and experimental practicality. Further work should help to determine which algorithms are most effective for identifying relevant kinases and pathways from inhibitor-induced perturbations in the phosphoproteome, for target deconvolution based on phenotypic screens, and for rational design of polypharmacological agents and drug combinations (Gujral et al., 2014b; Hernandez-Armenta et al., 2017; Tang, 2017; Rocca and Kholodenko, 2021) Finally, machine learning is poised to provide notable advances in determining features of kinase networks that predict drug efficacy for personalized medicine, as well as in these other areas.

In summary, in-depth knowledge of target engagement by chemical probes in cells, coupled with increasingly sophisticated data acquisition and analysis pipelines, suggest that pharmacological approaches to understanding context-specific kinase networks will continue to yield vital insights for the foreseeable future.

## Abbreviations

AML, Acute Myeloid Leukemia; BRET, Bioluminescence Resonance Energy Transfer; CETSA, Cellular Thermal Stability Assay; DSF, Differential Scanning Fluorimetry; EBDT, Expectancy of Being Downstream Target; KCGS, Kinase Chemogenomic Set; kiCCA, Kinobead Competition and Correlation Analysis; KiPIK, Kinase inhibitor Profiling to Identify Kinases; KiR, Kinome Regularization; KSEA, Kinase-Substrate Enrichment Analysis; KSR, Kinase-Substrate Relationship; MIBs, Multiplexed Immobilized Beads; PPI, Protein-Protein Interaction.
